# Unraveling ecosystem functioning in intertidal soft sediments: the role of density-driven interactions

**DOI:** 10.1038/s41598-020-68869-4

**Published:** 2020-07-17

**Authors:** Stefano Schenone, Simon F. Thrush

**Affiliations:** Institute of Marine Science, University of Auckland, Auckland, 1142 New Zealand

**Keywords:** Biogeochemistry, Ecosystem ecology, Marine biology, Ecosystem services

## Abstract

Although they only occupy a relatively small portion of the surface of the planet, coastal habitats are some of the most productive and valued ecosystems in the world. Among these habitats, tidal flats are an important component of many harbours and estuaries, but their deterioration due to human activities poses a serious threat to biodiversity and ecosystem function. Benthic communities are usually arranged in patches dominated by key species with overlapping distributions. Understanding the ecological consequences of interactions between these species in transition zones where their habitats overlap is necessary in order to quantify their contribution to overall ecosystem functioning and to scale-up and generalize results. Spatial transition in abundance and the interaction of multiple factors that drive ecosystem function are complex processes that require real-world research. Through a multi-site mensurative experiment, we show that transition areas drive non-linear effects on biogeochemical fluxes that have important implications for quantifying overall functioning. In our study the main drivers of ecosystem function were the abundance of two large but functionally very different species rather than biodiversity per se. Furthermore, we demonstrate that the use of the biogenic features created by specific infaunal species at the sediment–water interface is a better predictor of ecosystem functioning than the density of the species per se, making this approach particularly appealing for large scale, mapping and monitoring studies.

## Introduction

Coastal habitats only occupy about 10% of the ocean’s surface area but make a disproportionate contribution to key earth-system processes^1^. Humanity has benefited from and evolved around coastal ecosystems but this has come at a cost of massive exploitation and intense deterioration of these systems. Nevertheless, recent reviews of the global value of estuarine and coastal ecosystems highlight these ecosystems still deliver many critical ecosystem services^1–3^.

The global distribution of tidal flats occupies at least 127,921 km^2^^4^. These soft sediment environments are complex ecosystems containing strong physical gradients that affect the distribution of species and physico-chemical conditions. These features interact with biology resulting in patchy spatial distributions of communities and ecosystem functions across multiple spatial scales. Such patchiness is often not as apparent as in other ecosystems where above ground structures define patches (e.g. terrestrial and marine forests). This heterogeneity is a powerful indicator of ecological health but confounds the simple up-scaling of ecosystem function measurements and thus the estimate of ecosystem services at scales most relevant to society^5^. When patches dominated by specific community types overlap, they create interface areas where communities and habitat features grade into one another, with largely unexplored consequences for ecological functioning^6^. These areas of transition between contrasting patches of habitat can lead to interactive effects and emergent properties and therefore cannot be fully characterized by solely characterizing the adjacent patches^7^.

**Figure 1:**
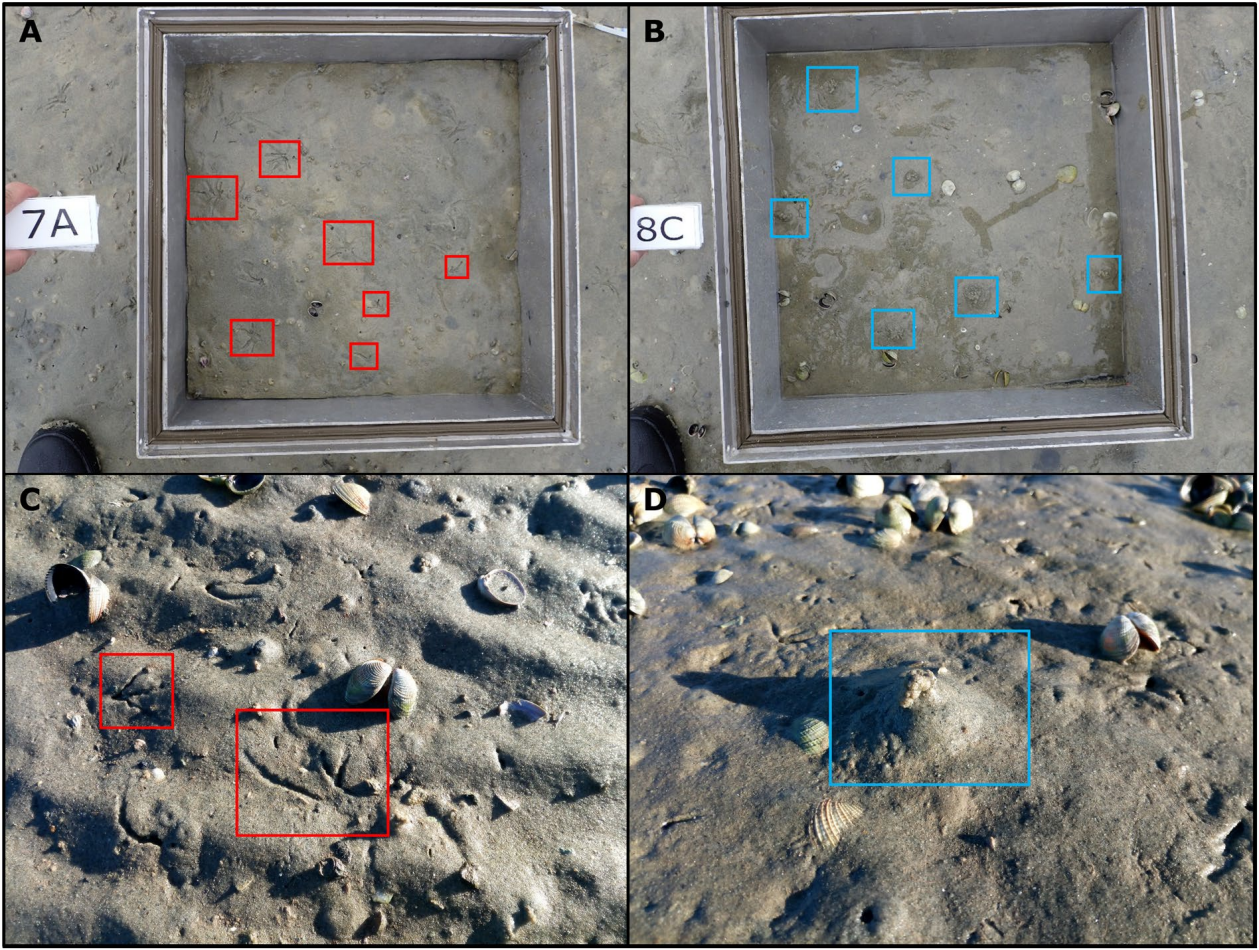
Examples of biogenic features counts. The top panels are examples of pictures of the sediment surface inside the experimental incubation chambers, showing in red the annotation of the feeding tracks of Macomona liliana (**A**) and in light blue that of the sediment mounds created by Macroclymenella stewartensis (**B**). In the bottom panels, close-up pictures of the biogenic features of both M. liliana (**C**) and M. stewartensis (**D**) are included for reference.

Due to the complexity of interactions involved in driving rates and processes in these heterogeneous marine sediments, empirical measurement is essential, but exceedingly challenging. To resolve this fundamental problem we focused on resolving the shifts in multiple ecosystem functions associated with two co-occurring and functionally important species that differentially influence a variety of sedimentary rates and processes^8^. Adult *Macomona liliana* (tellinid bivalve) are ecosystem engineers that alter the sediment and its biogeochemical properties, playing an important role in community dynamics and benthic fluxes^9^. The polychaete *Macroclymenella stewartensis* (maldanid) is a head-down conveyor belt feeder that feeds at depth in the sediment and defecates at the surface. As a result of their biological activity, both species create distinctive microtopographic features on the sediments surface providing opportunities to quickly assess major changes in abundance and identify how these species partition the habitat in the transition zones (Fig. 1). Using in-situ benthic incubation chambers and an organic matter degradation assay we measured the fluxes of dissolved oxygen (O_2_) and ammonium (NH_4_^+^) as well as the organic matter degradation rate at the sediment surface (C_o_) and the extinction coefficient of organic matter degradation with sediment depth (k). By quantifying these ecosystem functions at different locations with varying densities of target species, we were able to demonstrate how the co-occurrence of both species at high densities tends to decrease biogeochemical fluxes as compared to patches dominated by either species and how this negative effect changed with the relative density of the two organisms. These sandflat communities are species rich (c 100–150 macrofauna species) and yet, using a methodology originally developed to understand the variation in community data (“variance partitioning”^10^), we were able to tease apart the role of these large species that leave signatures on the sediment surface from the role of the community (biodiversity) in driving ecosystem function.

## Results

**Figure 2:**
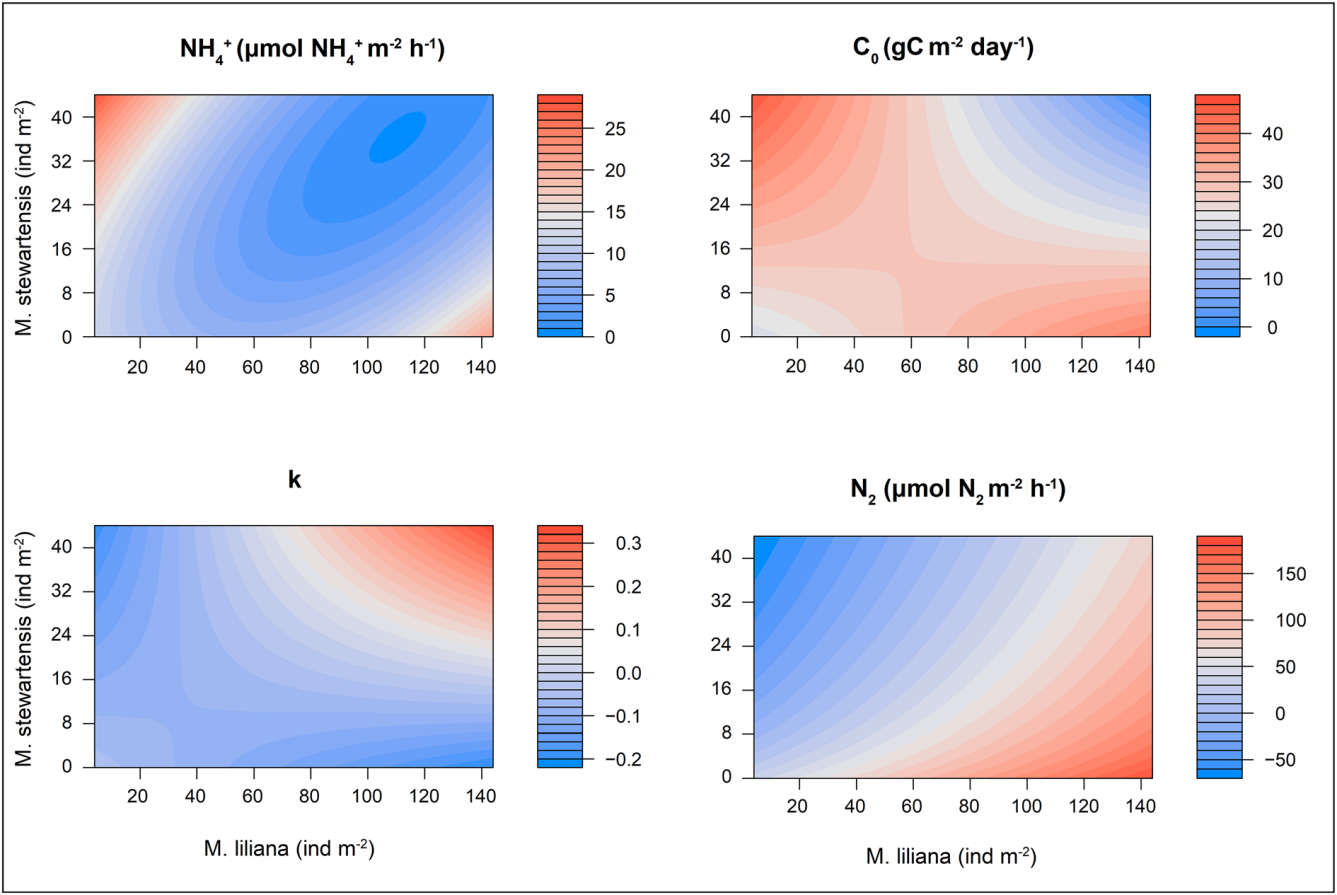
Effect of the interaction between M. liliana and M. stewartensis. Contour plots showing the modelled effect of the interaction between *M. liliana* and *M. stewartensis* on ecosystem functions as their densities change. Clockwise from upper left: ammonium efflux (NH_4_^+^), organic matter degradation at the sediment surface (C_o_), denitrification (N_2_), extinction coefficient of organic matter degradation with sediment depth (k). Note the scale is different for different functions.

Exploring the importance of the single biotic and abiotic components, each ecosystem function was driven by different variables (Table 1). However, the presence of *M. liliana* and *M. stewartensis* and their interaction were consistently important predictors, while the environmental variable that was most consistently retained in the models was sediment porosity. Our ecological functions models, except for O_2_ consumption and denitrification, identified an important interaction effect. Changes in the relative abundance of *M. liliana* and *M. stewartensis*, measured from the change in the density of their biogenic features, modified the nature of their effect on multiple ecosystem functions (Fig. 2). NH_4_^+^ efflux showed the highest rates when only one of the two species was present at the highest density. Lowest NH_4_^+^ efflux occurred when both organisms where present at the highest densities. Simultaneous, low densities of both organisms also led to low NH_4_^+^ efflux. Both organic matter degradation parameters showed a similar response to the interaction, with high C_o_ and low k present in areas of high densities of *M. liliana* with and low densities of *M. stewartensis* and vice versa, and low C_o_ and high k when both organisms were present at high density simultaneously. Finally, even though backwards variable selection retained the density of both organisms but not the interaction term in the final statistical model explaining denitrification, we investigated the changes in denitrification rates with changing densities of *M. liliana* and *M. stewartensis*. Denitrification was highest when the densities of *M. liliana* were highest and those of *M. stewartensis* lowest. An increase in *M. stewartensis*, or a decrease in *M. liliana* densities, led to a decrease in net denitrification rates. Net nitrogen fixation was predicted at average to high densities of the polychaete in presence of the lowest densities of the bivalve.

**Table 1:** Significant variables for each one of the five ecosystem functions measured.

Factor	N_2_	NH_4_^+^	O_2_	C_o_	k
*M. liliana*	14.78	24.41	10.28	0.14	3.47
*M. stewartensis*	12.32	0.46		7.75	20.73
*M. liliana* x *M. stewartensis*		8.51		19.37	24.78
Porosity	7.14	22.62	21.16	17.92	3.05
Mud %		6.82			
Grain size		5.31	12.91		16.65
Organic matter		7.01	2.44		
Total explained	34.24	75.14	46.8	45.18	68.88

Important variables for each predictive model—identified by backwards variable selection—and their relative importance (%).

**Figure 3:**
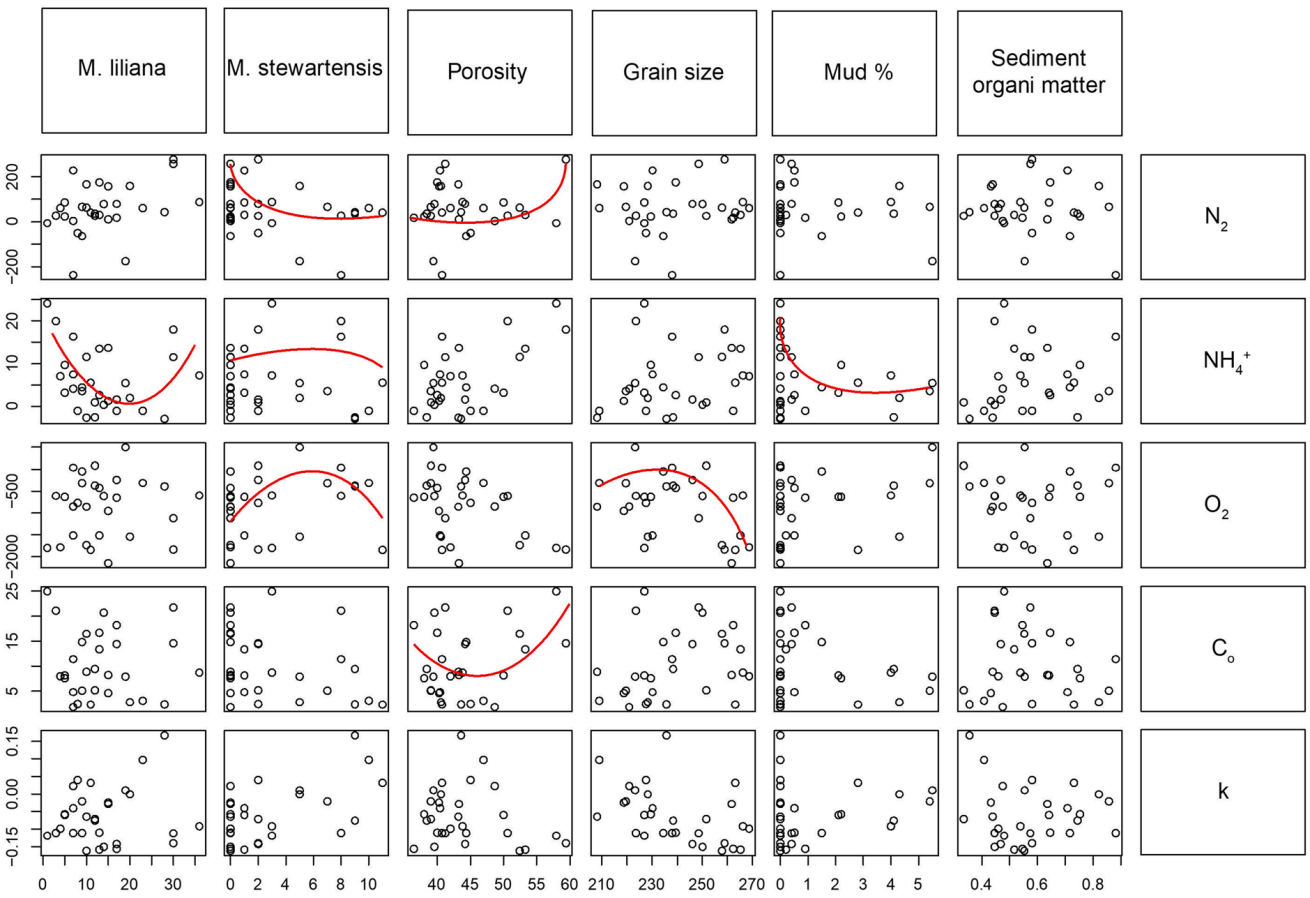
The form of the non-linear relationships. The red lines represent the shape of the non-linear relationships between biological and environmental variables (on the top) and the ecosystem functions (on the right). Where the red line is missing, the relationship was either linear or not significant.

Although best results were obtained using untransformed data, all models included significant polynomial terms emphasising non-linear rates across the sandflats. Organic matter degradation seemed to be mainly driven by linear relationships and the only non-linear relationship of organic matter degradation at the sediment surface (C_o_) was with porosity (Fig. 3). Denitrification, NH_4_^+^ efflux and O_2_ consumption had different non-linear predictors, however *M. stewartensis* showed a non-linear relationship with ecosystem functions more consistently than the other predictors.

**Figure 4:**
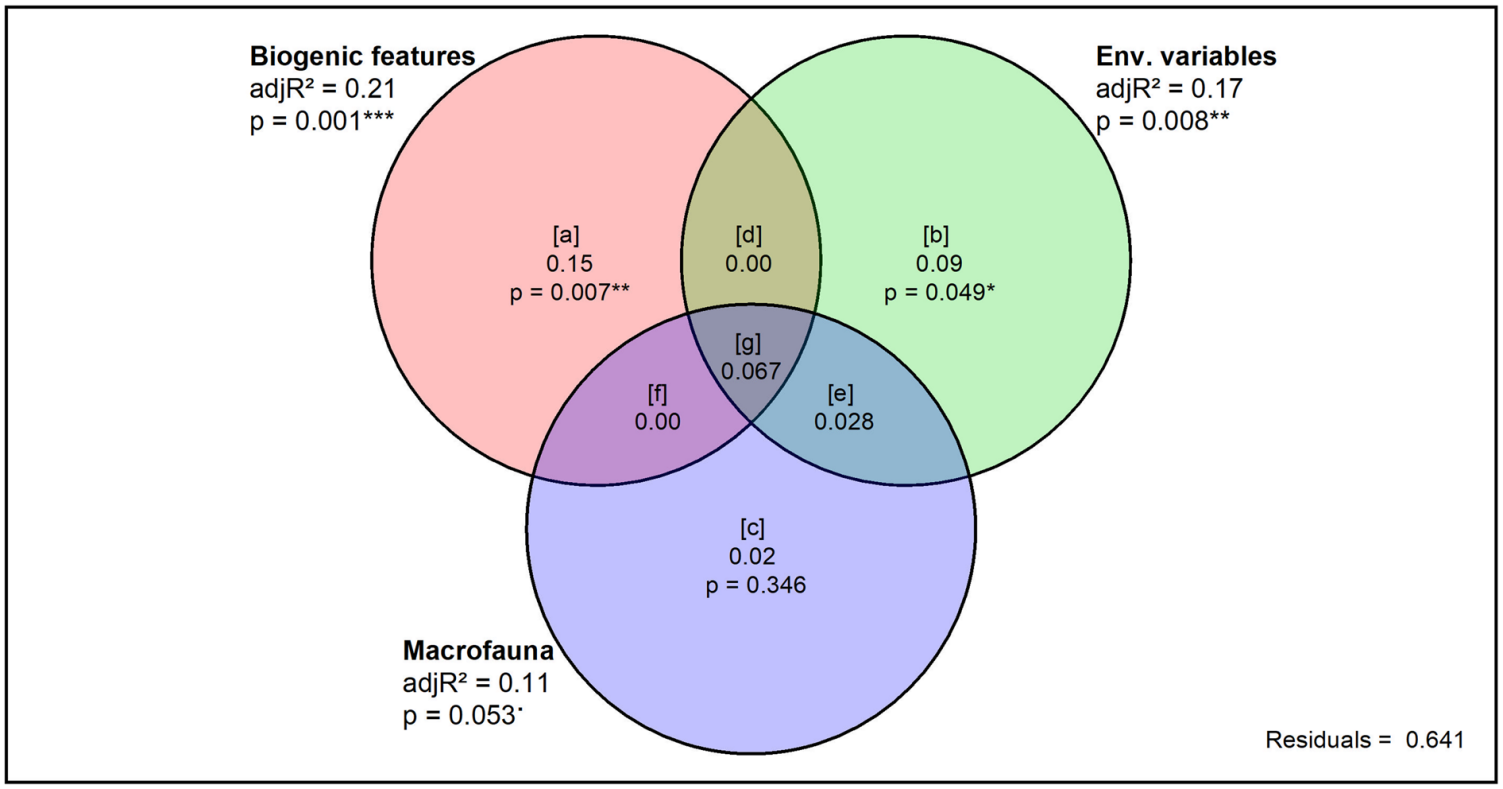
Variance partitioning. Results of the variance partitioning of the functions data between biogenic features of *M. liliana* and *M. stewartensis*, environmental variables and the rest of the macrofaunal community. [a] is the fraction explained purely by *M. liliana* and *M. stewartensis*, [b] is the fraction explained purely by environmental variables, [c] is the fraction explained purely by the rest of the macrofaunal community and [d], [e], [f], [g] are the fractions of explained variance shared by two or more of the sets of variables. The adjR^2^ values indicate the total variance explained by a set of variables. Note the size of the circles is not correlated with the variance explained.

Partitioning the variation in the ecological functions between the biogenic features of *M. liliana* and *M. stewartensis*, the measured environmental variables and the rest of the macrofaunal community showed that biogenic features explained the largest portion (21%, p = 0.001) of variance, followed by the environmental variables (17%, p = 0.008) and macrofauna (11%, p = 0.053) (Fig. 4). These biogenic features are signatures on the sediment surface of the activity of Macomona (feeding traces) and Macroclymenella (faecal mounds) and offer an estimate of their abundance and biological activity. However, most of the variation explained by macrofauna was shared with the biogenic features and environmental variables, while the non-shared portion explained purely by macrofauna accounted for only 2% of the total variation (p = 0.346). Consistently, when we analysed the variance partitioning of one function at a time, macrofauna was only important in explaining NH_4_^+^ efflux, the organic matter extinction coefficient (k) and surface organic matter degradation (C_o_) (23, 21 and 16% respectively). The non-shared portion of variance purely attributable to macrofauna was not significant for any of these functions.

## Conclusions

Our results confirmed the importance of specific, large but functionally different, species for ecosystem functioning and in particular, highlighted the importance of studying transition zones where their interaction can significantly alter benthic fluxes. Looking at the two species individually, our findings were consistent with previous studies on their effect on biogeochemical fluxes^11–14^. However, the presence of non-linear relationships and significant interactions indicates that these effects can change with the density of the organisms. As hypothesized based on our previous mesocosm experiments^8^, the co-occurrence of both species at high densities reduced fluxes. However, this negative effect changed with the relative density of the two organisms and became positive when one of the two species was dominant. Several studies report the positive effects of *M. liliana* on nitrogen fluxes and primary production^9, 15,16^. However, our findings suggest that such effects can be completely negated in transitional areas with high densities of *M. stewartensis*. These transition zones are a common component of the tidal flat landscape and can occupy a vast portion of the ecosystem^17–20^, therefore exerting an important influence on overall ecosystem functioning at large scales. Tellinid bivalves and maldanid worms are cosmopolitan organisms that inhabit intertidal and subtidal marine soft-sediments all over the world^15,21–23^. Although, different systems are likely to host different sets of key species, with different functional traits, the implications of the results of our experiment are of global importance in linking ecosystem function measurements to the mapping of ecosystem services. The latter is often very coarse scale with little consideration for the spatial or temporal variation in function. Our results, with a 200-fold change in functions across intertidal flats, highlight the need for much quantification of the drivers of ecosystem function.

The unprecedented and pioneering use of variance partitioning in biodiversity-ecosystem functioning research allowed us to distinguish between the impact of the whole macrofaunal community and that of our surface feature-forming species. Our findings indicate that even though community composition is important in explaining ecosystem functioning, our two target species held more explanatory power than the rest of the macrofaunal community. This is consistent with the “passengers and drivers” model, which proposes that in most ecosystems certain species have a disproportionate ecological impact (“drivers”) while others have a negligible effect (“passengers”)^24^. However, while all the functions that we measured are linked to sediment biogeochemistry, driver and passenger species may be different when the entire multifunctionality of sediments is considered. Our results suggest that the sampling or the entire macrofaunal community is not always necessary. While our models generally identified *M. liliana* and *M. stewartensis* as important variables to explain fluxes, the variance partitioning showed that macrofaunal community only explained a small portion of the variation in ecosystem functions and most of the explained variation was shared with (i.e. already explained by) the other sets of variables. Therefore, the sampling of surface features of key species and a few easily measurable environmental variables can be sufficient to predict ecosystem functioning, which makes this approach particularly appealing for large-scale, mapping and monitoring studies.

In this context, our study also demonstrates the potential of using surface features to scale up ecosystem functioning measurements. The density of the small biogenic surface features, in fact, is not only a good surrogate for species distribution and density but also contains an intrinsic measure of the activity of the infaunal organisms, which in return affects ecosystem functions^25^. More active (e.g. feeding, excreting) individuals indeed have a bigger impact on sediment biogeochemistry than less active ones and this results in a greater number of biogenic features. Counting biogenic structures is not only non-invasive and faster than sampling macrofauna but can easily be applied over large scales through remote sensing.

Our analysis opens up new ways to use the information provided by functionally important species and their surface features to up-scale ecological processes measurements and map ecosystem functions at the landscape level. We indeed need more research to focus on the quantification of ecosystem services at scales that are relevant to society and on the underlying role of living organisms in the provision of these services.

## Methods

### Study location and sampling design

Field sampling took place in the intertidal zone of the Whangateau Harbour, New Zealand, in April 2018. The extensive intertidal flats are predominantly composed of medium to coarse grain sand with a relatively low percentage of mud (< 6%). Sampling was conducted at four sites in different parts of the harbour (“Tramcar Bay”, 36°18.59′S, 174°46.71′E; “Lews Bay”, 36°18.72′S, 174°46.42′E; “Horseshoe Island”, 36°19.02′S, 174°46.17′E and “Point Wells”, 36°19.21′S, 174°45.59′E). 30 stations distributed across sites to maximise information at different scale were sampled. Sampling covered a wide range of densities of both target species and encompassed patches dominated by each species and transitional areas.

### Benthic flux measurements

To measure the changes in the concentration of solutes we used opaque benthic incubation chambers and rapid organic matter assay (ROMA). At each sampling station we deployed one dark benthic chamber as described in^26^. The chambers incubated a volume of approximately 30 L of sea water and the incubations lasted for approximately 4 h, during high tide. Water was sampled from the chambers at the beginning and the end of the incubation period. We deployed dark, 1 L plastic bottles filled with ambient sea water, secured to the sediment surface in proximity of the chambers. Ambient water external to the chambers was also sampled at the beginning and the end of the incubations. Samples for O_2_ and N_2_ were transferred into 12 mL glass vials and stored in a portable ice chest until stored in a fridge. Samples for dissolved inorganic nitrogen (DIN; NH_4_^+^+NO_2_^−^+NO_3_^−^) were pressure-filtered through a Whatman GF/C glass fibre filter into 50 mL polyethylene centrifuge tubes and kept on ice prior to freezing. Since NO_2_^−^, NO_3_^−^ and PO_4_^3−^ levels were close to the detection limit of the instruments, only NH_4_^+^ was used in the statistical analysis of DIN fluxes.

For the ROMA, 10 days prior to the incubations one ROMA plate was deployed at each sampling station (see^27^ for a description of the methodology). The plates were then incubated in the sediment for 10 days prior to the flux measurements, then collected and stored in a portable ice chest until they were analysed on the same day.

### Sediment and macrofauna

At low tide, before the incubations, sediment characteristics were sampled next to each chamber. Benthic macrofauna was sampled at each sampling station (1 × 13 cm dia. × 15 cm deep cores), sieved over a 500 µm mesh and preserved in 70% isopropyl alcohol. Specimens of *Austrovenus stutchburyi* and *Paphies australis* in the samples were counted in the field and returned to the sediment alive due to local restrictions on their harvesting. Using a tripod to maintain a constant angle and distance, the sediment surface contained within each chamber base was vertically photographed to count *M. liliana* feeding tracks and *M. stewartensis* sediment mounds. After the incubations the sediment contained in the chambers was excavated to a depth of 15 cm and sieved over an 800 µm mesh to count the total number of *M. liliana* and *M. stewartensis* individuals.

### Laboratory analyses

O_2_ and N_2_ concentrations were determined by membrane-inlet mass spectrometry (MIMS) with a Pfeiffer Vacuum QMS 200 quadrupole mass spectrometer^28^. DIN concentrations were determined by flow injection analysis (FIA) with a Lachat Quick-Chem 8000 automated ion analyser^29^. Sediment porosity and organic matter content were determined from dried (48 h at 60 °C) and ashed (4 h at 500 °C) sediment samples respectively. Sediment grain size was measured with a Malvern Mastersizer-S. Preserved and stained macrofaunal samples were sorted under a dissecting microscope. All organisms were counted and identified to the lowest possible taxonomic level (usually species). Carbon consumption was measured by the change in agar volume in each well on the ROMA plate using an agar-to-carbon conversion factor of 0.026. Using linear regression to analyse the relationship between the natural log of organic matter degradation rate and the depth of the wells, we were able to calculate two parameters: the organic matter degradation rate at the sediment surface (C_o_) and the extinction coefficient (k)^27^.

### Image analysis

The density of biogenic features within the surface area delimited by the experimental chambers was calculated from the pictures taken before the start of the benthic flux incubations. Features of both *M. liliana* and *M. stewartensis* were manually counted.

### Data analysis

**Figure 5:**
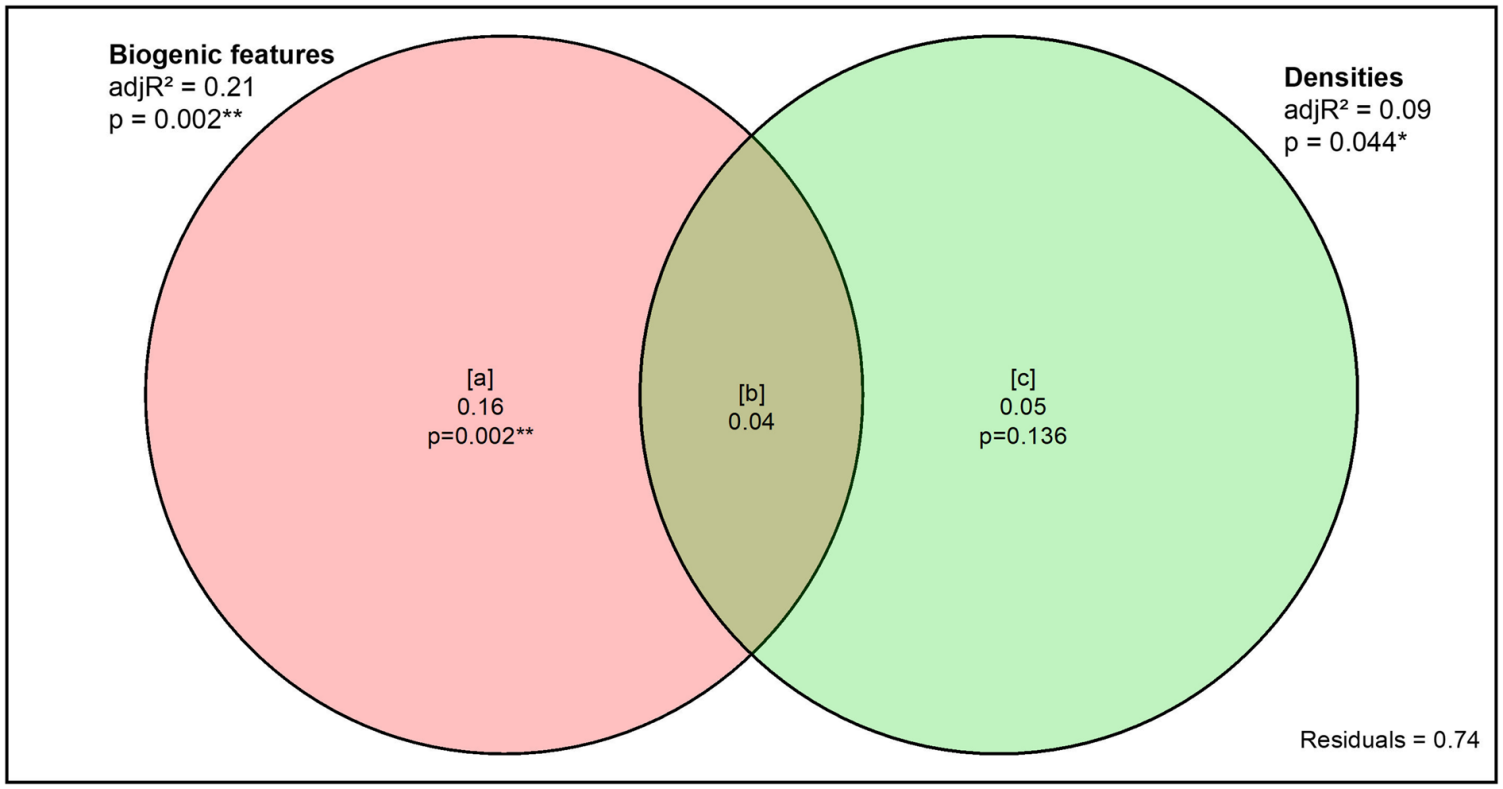
Biogenic features vs. species density. Variance partitioning of the multivariate functions data between the density of surface biogenic features and actual density of *M. liliana* and *M. stewartensis*.

The relationship between surface features and actual densities of *M. liliana* and *M. stewartensis* in the sediment was assessed via linear regression. Variance partitioning^10,30^ was then computed using the R ‘vegan’ package to compare the portion of the multivariate functions data explained by the density of surface features and the density of target species, respectively. The significance of each portion was tested using Redundancy Analysis (RDA). *M. liliana* and *M. stewartensis* densities were highly correlated with the densities of surface features they produced (R^2^ = 0.74 and 0.88 respectively). However, using variance partitioning we distinguished between how much of the variation in functioning was explained by the density of biogenic features compared to that explained by the actual density of *M. liliana* and *M. stewartensis* in the sediment. The former explained 21% of the variation in the functions data while the latter only explained 9% (Fig. 5). Therefore, we chose the densities of biogenic features produced by *M. liliana* and *M. stewartensis* over their actual densities in our analysis.

The variables were then divided into three sets of variables: “biogenic features”, (i.e. the density of the surface features of *M. liliana* and *M. stewartensis*); “env. variables”, (i.e. all the sampled environmental variables); “macrofauna”, (i.e. the information on the macrofaunal community, excluding *M. liliana* and *M. stewartensis*). Using variance partitioning, we calculated how much of the variability in multifunctionality (i.e. the entire set of functions) was explained by each set of variables. To explore the differences in the partitioning of the variation for each single function we then used partial linear regression on denitrification, NH_4_^+^ efflux, O_2_ consumption and organic matter degradation separately.

Generalised linear modeling with incorporated nonlinearities and backwards variable selection was then used to determine which of the biotic and abiotic variables better predicted ecosystem functions. Best models were selected based on the residual by predicted plots, residual normal plots and partial leverage plots. For each model, we determined the relative importance of each predictor variable using the lmg metric in the ‘relaimpo’ R package^31^. When the models identified significant interactions, two-dimensional contour plots were created to investigate the effect of the interaction on the ecosystem function of interest. All statistical analyses were performed with R v3.6.1^32^.

## Data Availability

The data that support the findings of this study are available from the corresponding author upon reasonable request.
